# Organ-specific proteome analysis for identification of abiotic stress response mechanism in crop

**DOI:** 10.3389/fpls.2013.00071

**Published:** 2013-04-03

**Authors:** Setsuko Komatsu, Zahed Hossain

**Affiliations:** ^1^National Institute of Crop Science, National Agriculture and Food Research OrganizationTsukuba, Japan; ^2^Department of Botany, West Bengal State UniversityKolkata, India

**Keywords:** crop, proteomics, organ-specific, abiotic stress, flooding, drought, salinity

## Abstract

Abiotic stresses, such as flooding, drought, salinity, and high/low temperatures, are the major constraints that global crop production faces at present. Plants respond to a stress by modulating abundance of candidate proteins, either by up-regulating expression or by the synthesizing novel proteins primarily associated with plant defense system. The cellular mechanisms of stress sensing and signal transduction into cellular organelles have been reported. Nevertheless, the responses of plant cells to abiotic stresses differ in each organ. As the correlation between the expression of mRNAs and the abundance of their corresponding proteins is difficult to assess in specific organs, proteomics techniques provide one of the best options for the functional analysis of translated regions of the genome. The present review summarizes the organ-specific proteome analyses for better understanding of the response mechanisms of crops to abiotic stresses, including flooding, drought, and salinity. The differential organ-specific responses against each of these stresses are discussed in detail to provide new insights into plant stress response mechanisms at protein level.

## Introduction

Abiotic stress is a key limiting factor that impairs growth and yield of agricultural crops around the world (Hossain et al., 2012). Stressful environment may lead to delay in seed germination, reduced seedling growth, and finally decreased crop yield. Under abiotic stress, various compounds are either synthesized in the roots such as hormones and amino acids and/or taken up such as water and mineral nutrients by the roots and must be transported to the shoot to enable normal leaf functioning (Ghanem et al., 2011). Proteins associated with the primary function of an organ, are uniquely expressed in that specific organ/tissue (Watson et al., 2003). This organ-specific expression of proteins is thus essential for plant growth and development. Organ-specific proteomics analyses help us in better understanding the response mechanisms of plants toward abiotic stresses.

In higher plants, leaves represent highly specialized organ that is primarily engaged in photosynthesis. It has been shown in several reports that rates and activities of photosynthesis are highly dependent on the development and age of the leaf, and this is also correlated with the accumulation of proteins such as ribulose-1,5-bisphosphate carboxylase/oxygenase (RuBisCO) and other photosynthesis-related proteins (Maayan et al., 2008; Urban et al., 2008). Leaves also play major role in transporting essential elements and water from the roots to aerial parts. Plant roots can sense gravity, water, nutrients, and other signals in the soil. Moreover, they have the ability to secrete numerous compounds that protect the root apex and regulate root growth (Feldman, 1984; Aiken and Smucker, 1996). A better understanding of the mechanisms determining root length and branching is expected to improve crop production/yields (Lynch, 2007). Abiotic stress initially affects the underground or overground part of a plant and is sensed by the roots or leaves that finally trigger cellular signal transduction pathways leading to molecular and metabolic changes. Hence, for better understanding of how plants respond and adapt to abiotic stresses, it is important to focus on the root and leaf system.

Analysis of organ-specific protein abundance provides rich information about the response mechanisms of plants to abiotic stress. However, total protein extraction from leaves is difficult due to the presence of abundant proteins, which also interfere with the expression analysis of low-abundance proteins by physically or chemically masking their abundance. In contrast to leaves, the concentration of proteins in roots is relatively low, also making protein identification difficult. The direct homogenization of plant organs with a solubilization buffer has not yielded high-quality proteome maps for leaves or roots, suggesting that the direct extraction method is not suitable for plant proteins. Hence, more research needs to be focused on the improvement of protein extraction methods for obtaining high resolution organ based proteome map.

## Protein extraction from organs

To magnify the expression of low-abundance proteins, the elimination of high-abundance proteins from samples is compulsory. Using a polyethylene glycol (PEG)-fractionation method, RuBisCO, which is an abundant protein in the leaf, was eliminated from other leaf proteins during extraction (Ahsan et al., 2007a). In this method, proteins were first extracted from tomato leaves using Mg/Nonidet P-40 buffer consisting of 0.5 M Tris-HCl, 2% Nonidet P-40, 20 mM MgCl_2_, 2% 2-mercaptoethanol, 1 mM phenyl methyl sulfonyl fluoride, and 1% polyvinyl polypyrrolidone, and were then fractionated with 15% PEG (Ahsan et al., 2007a). Hashimoto and Komatsu (2007) reported the preparation of an anti-RuBisCO LSU antibody-affinity column with protein A-Sepharose as a resin. The leaf protein extract was incubated with purified IgG, and was further incubated with rehydrated protein A-Sepharose resin. The resin was then added to a Spin X cup (Pierce, Rockford, IL, USA), centrifuged, and washed several times with the wash solution. Because the collected flowthrough from the wash solution contained released IgG molecules, these solutions were applied to the Protein A Agarose kit column (KPL, Gaithersburg, MD, USA) to trap the released IgG, thereby removing the extra IgG molecules (Hashimoto and Komatsu, 2007).

For root proteomics studies, a number of enrichment methods have been used due to presence of high levels of low-abundance proteins in roots. The most widely used protein extraction method is trichloroacetic acid (TCA)/acetone precipitation. Using this method, Nanjo et al. (2012) extracted proteins from roots with a solution of 8 M urea, 2 M thiourea, 5% CHAPS, and 2 mM tributylphosphine. Ahsan and Komatsu (2009) reported that treatment of root with Mg/Nonidet P-40 buffer followed by extraction with alkaline phenol and methanol/ammonium acetate produced high-quality proteome maps consisting of numerous well-separated spots with high intensity, on two-dimensional polyacrylamide gel electrophoresis (2-DE) gels.

Various protein extraction and solubilization methods have been evaluated for obtaining high-quality, reproducible proteome reference maps of various plant organs. For example, when seed proteins of soybean were extracted by TCA/acetone precipitation, substantial horizontal streaking was observed in 2-DE gels (Mooney et al., 2004). This result suggests that the TCA/acetone precipitation method does not completely remove non-protein contaminants from plant organs (Komatsu and Ahsan, 2009). Although both TCA/acetone- and phenol-based methods are reliable and efficient methods for extracting proteins from various plant organs (Rose et al., 2004; Espagne et al., 2007), experimental evidence for various types of soybean organs has demonstrated that the phenol-based method gives reproducible, high-quality proteome maps compared to other available methods (Ahsan and Komatsu, 2009). In recent time, gel-free proteomics techniques are often being used for proteome analysis; however, the purification of protein extracts using methods such as TCA/acetone precipitation is needed.

## Leaf proteomics analysis under abiotic stress

### Leaf proteomic analysis under flooding stress

The negative impacts of flooding include inhibition of leaf growth, reduction of biomass production, and ultimately, reduced seed yield. Flooding can also result in reduced stomatal conductance and decreased chlorophyll a and b content in leaves (Gomes and Kozlowski, 1980). The decrease in chlorophyll content was more significant in older leaves, suggesting that chlorophyll degradation proceeds more rapidly in leaves that are closer to flooded roots. The reduction of plant biomass in response to flooding may be directly related to stomatal limitations on net photosynthesis that result in reduced carbon assimilation (Mielke et al., 2003). Restriction of photosynthetic activity may also be influenced by changes in the components of the biochemical reactions, such as RuBisCO and other photosynthesis-related proteins (Maayan et al., 2008). Proteomics analyses of leaf tissue have revealed that the majority of the identified proteins are involved in energy production and primary/secondary metabolism (Donnelly et al., 2005).

Ahsan et al. (2007a) examined protein changes in tomato leaves exposed to waterlogging stress and found that the expression of proteins associated with stress/defense mechanisms and energy/metabolism were increased, while photosynthesis- and protein biosynthesis-related proteins were decreased. Among the identified photosynthesis-related proteins, the expression of RuBisCO was decreased in the total soluble proteins in response to waterlogging stress. RuBisCO has a dual function: it acts as a carboxylase mediating photosynthetic CO_2_ assimilation and as an oxygenase catalyzing the first step of the photorespiratory pathway. Waterlogging stress-induced decrease in abundance of RuBisCO activase protein has also been reported in tomato leaves (Ahsan et al., 2007a) that maintains RuBisCO in an active, functional state. In addition, the authors reported that submergence stress induced the formation of reactive oxygen species (ROS) that ultimately leads to degradation of the subunits of RuBisCO and RuBisCO activase (Ahsan et al., 2007a). Inhibition of protein biosynthesis and activation of proteases in the tomato leaves were found to be the major causes of injuries due to waterlogging. In addition, increased expression of heat shock proteins and other stress-related proteins was also observed, indicating that activation of the defense system promoted survival under submerged conditions (Ahsan et al., 2007a).

Khatoon et al. (2012) investigated the organ-specific response mechanism in 1-week-old soybean seedlings under flooding stress by analyzing protein profiles in the roots, hypocotyls, and leaves using a gel-based proteomics technique. Among a total of 577 protein spots identified in leaves, 24 and 26 spots were increased and decreased, respectively, in response to flooding stress. Compared to untreated seedlings, 16 protein spots exhibited more than a two-fold change, with 6 spots increasing and 10 spots decreasing. In leaves, more metabolism-related, cytoplasmic, and chloroplastic proteins were decreased than were increased, while all of the disease/defense-related proteins were decreased. As compared to the roots and hypocotyls, fewer energy-related proteins were observed in leaves. Among the reduced metabolism-related proteins, isoflavone reductase, which plays an essential role against oxidative injuries, was identified. Isoflavone reductase is involved in the biosynthesis of alkaloids that play important roles in defense against various stresses (Kajikawa et al., 2009). The reduced levels of isoflavone reductase not only in leaves, but also in roots, is one of the factors involved in the decreased efficiency of the antioxidant system in soybean seedlings exposed to flooding stress. The decrease of isoflavone reductase and several other disease/defense-related proteins in the roots and leaves of flooded seedlings compared to non-stressed seedlings indicates that the defense response is highly suppressed in soybean seedlings under flooding stress.

### Leaf proteomic analysis under drought stress

Stomatal closure in response to drought stress primarily results in a reduced rate of photosynthesis. Environmental conditions that increase the transpiration rate also tend to increase the pH of leaf sap that in turn, promotes abscisic acid accumulation and lead to reduced stomatal conductance (Davies et al., 2002; Wilkinson and Davies, 2002). Very severe drought conditions limit photosynthesis due to a decline in RuBisCO activity (Bota et al., 2004). The activity of the photosynthetic electron transport chain is finely tuned to the availability of CO_2_, and photosystem II activities often declines in parallel under drought conditions (Loreto et al., 1995). Proteomic analyses of leaves from rice (Salekdeh et al., 2002a,b; Ali and Komatsu, 2006; Ke et al., 2009), sugar beet (Hajheidari et al., 2005), wild watermelon (Yoshimura et al., 2008), tall wheatgrass (Gazanchian et al., 2007), *Quercus ilex* (Echevarría-Zomeno et al., 2009), *Populus euramericana* (Bonhomme et al., 2009), wheat (Caruso et al., 2009), sunflower (Castillejo et al., 2008), and soybean (Mohammadi et al., 2012a,b) have identified numerous drought-responsive proteins, which were chiefly involved in redox regulation, oxidative stress response, signal transduction, protein folding, secondary metabolism, and photosynthesis. Vincent et al. (2005) used a proteomics approach to understand the impact of drought stress on the lignification of maize leaves, and revealed that proteins involved in lignification and flavonoid synthesis have an important contribution to the plant response to water deficit.

### Leaf proteomic analysis under salinity stress

Soil salinity is an ever-present threat to crop yield, especially in arid and semi-arid zones. Salinity, chiefly due to the presence of NaCl, is considered the single most widespread soil toxicity problem of global crop production (Hossain et al., 2006). On exposure to salt stress, expression of most of the photosynthesis-related proteins in leaves were found to be decreased, suggesting that NaCl adversely affects photosynthesis and energy production, and consequently reduces plant growth. Proteomic analyses of leaf tissue from rice (Salekdeh et al., 2002a,b; Abbasi and Komatsu, 2004), citrus (Tanou et al., 2009), glasswort (Wang et al., 2009), *Suaeda aegyptiaca* (Askari et al., 2006), and soybean (Sobhanian et al., 2010) have identified a variety of salt-responsive proteins. Among these proteins, abundance of calreticulin, a calcium-binding chaperone protein that plays a pivotal role in regulating calcium homeostasis and protein folding in the endoplasmic reticulum (Menegazzi et al., 1993; Wang et al., 2004), was decreased in rice leaves under osmotic stress (Zang and Komatsu, 2007). This finding indicates that calcium is an important secondary messenger in rice seedlings exposed to salt stress. The principal role of RuBisCO activase is to release inhibitory sugar phosphates, such as ribulose-1,5-biphosphate, from the active sites of RuBisCO to allow its activation by CO_2_ through carbamylation (Jordan and Chollet, 1983). Additionally, RuBisCO activase functions as a chaperone during stress (Rokka et al., 2001). In rice leaves, the levels of RuBisCO activase were decreased upon exposure to NaCl. This might be the prime reason of declined photosynthetic activity under NaCl stress (Parker et al., 2006). Dysfunction of protein is a common consequence of abiotic stress. Molecular chaperones/heat-shock proteins are responsible for proper protein folding, assembly and translocation (Wang et al., 2004). Although most newly synthesized proteins can fold in the absence of chaperones, a minority strictly requires assistance of these specialized proteins (Horvath et al., 2008). The 20-kDa chaperonin functions as a co-chaperone, along with cpn60, and in certain cases is essential for the discharge of biologically active proteins from cpn60 (Bertsch et al., 1992). Sobhanian et al. (2010) reported abundance of a 20-kDa chaperonin was increased in the leaves of soybean seedlings exposed to salt stress, suggesting the protective roles of chaperones in preventing the misfolding of proteins under salt stress. In addition, a decrease of the 50S ribosomal subunit, which catalyzes the peptidyl-transfer reaction of messenger RNA-directed protein biosynthesis (Kotusov et al., 1976), indicates that NaCl has an inhibitory effect on soybean protein biosynthesis and presumably leads to the observed reduction in plant growth under high salinity.

## Root proteomic analysis under abiotic stress

### Root proteomic analysis under flooding stress

Proteomic analyses of root including hypocotyl of 2-day-old soybeans exposed to flooding stress revealed that proteins related to glycolysis including UDP-glucose pyrophosphorylase and fructose-bisphosphate aldolase, disease/defense-related proteins such as ROS scavengers, chaperones, hemoglobin, and/or acid phosphatase, were highly affected (Hashiguchi et al., 2009; Komatsu et al., 2009; Nanjo et al., 2010). The flooding response mechanism in soybean during late growth stages was investigated in 3-week-old soybean seedlings subjected to flooding for 3 and 7 days (Alam et al., 2010). The study revealed that levels of enzymes of the glycolysis and fermentation pathways were mostly affected. This finding suggests that soybean seedlings respond similarly to flooding stress during early and late growth stages.

Kong et al. (2010) investigated the response mechanism of 2-day-old wheat roots exposed to flooding stress. The observed decrease in proteins involved in glycolytic pathway supports the notion that reduced carbohydrate metabolism and energy consumption are the primary responses that plants use to cope with flooding stress. In addition, proteins related to disease/defense were increased in abundance, while cell wall structure/modification-related proteins, including methionine synthase, were decreased, suggesting that cell growth was restricted to save energy in the unfavorable environment imposed by flooding. Haque et al. (2011) examined the response mechanism of wheat to flooding stress at different root depths, and found that proteins showing increased expression were related to energy and redox status, defense responses, and cell wall turnover. Based on the proteomic data, it was suggested that these proteins were possibly involved in alternative respiration and cell degeneration for promoting metabolic adjustment in response to the hypoxia stress-induced by flooding. Ahsan et al. (2007b) reported that tomato plants could cope with the severe conditions under submergence. In particular, proteins related to secondary metabolite biosynthesis, programmed cell death, and diseases/defense were increased in flooded tomato roots (Ahsan et al., 2007b). Metabolic adjustment for the management of energy consumption during cellular processes was the key adaptive response in tomato roots exposed to submergence stress, as evidenced by the increase of alcohol dehydrogenase and enolase, and decreased level of pyruvate dehydrogenase (Ahsan et al., 2007b). Proteomics based screenings of proteins conferring tolerance against submergence has provided novel information for future development of genetically engineered flood-tolerant crops.

### Root proteomic analysis under drought stress

Root development is strongly influenced by adverse growing conditions; however, root growth is typically less affected by drought stress than shoot growth (Franco et al., 2011). Thus, a decrease in the shoot: root ratio is commonly observed under drought stress, and results from either increased root growth or from a relatively larger decrease in shoot growth compared to root growth. In addition, roots typically contain a greater percentage of fine roots, which are capable of penetrating smaller soil pores and presumably optimize the exploratory capabilities of the root system as a whole, and also may have an important role in survival against drought. Proteomic analysis of the root in rice (Mirzaei et al., 2012), soybean (Toorchi et al., 2009; Alam et al., 2010; Mohammadi et al., 2012a), and *Brassica napus* (Damerval et al., 1988; Mohammadi et al., 2012b) under drought stress has identified a wide range of proteins, that lead to a better understanding of the mechanism of drought stress tolerance in plants.

Mirzaei et al. (2012) investigated how rice root systems in heterogeneous soils adapt to drought by comparing root tissues under four conditions: (1) fully watered; (2) fully droughted, and split-root systems where (3) one-half was watered and (4) the other half was droughted. Label-free proteomic analysis of these four kinds of roots resulted in the identification of 1487 non-redundant proteins, with nearly 900 proteins present in each treatment. Drought caused marked changes in expression, most notably in partially droughted roots, in which the levels of 38% of proteins were altered compared to adjacent, watered roots. In response to drought, pathogenesis- related proteins were generally increased. In contrast, heat-shock proteins were not detected in roots of fully watered plants. Proteins involved in transport and oxidation-reduction reactions were also highly dependent upon drought signals, with transport-related proteins largely absent in roots receiving a drought signal while oxidation-reduction proteins were typically increased during drought. The comparison showed that nine tubulins were strongly reduced in droughted roots, while the levels of six chitinases were increased, even when the signal arrived remotely from adjacent, droughted roots. This label-free proteomic analysis of water stress in split-root systems of rice has provided novel molecular insights into the heterogeneous translation patterns that occur in wet and dry soil zones.

### Root proteomic analysis under salinity stress

Root represents the vital plant organ that first encounters salt stress. Some salt stress-responsive genes and proteins are strongly induced in roots than in other organs (Yan et al., 2005). Proteomic analyses of the root in soybean (Sobhanian et al., 2010), rice (Liu et al., 2012), wheat (Guo et al., 2012), maize (Zörb et al., 2010), barley (Sugimoto and Takeda, 2009), and potato (Aghaei et al., 2008) under salt stress have been reported. These studies have revealed that a number of salt stress-responsive genes are more strongly induced in roots than in other organs.

Sobhanian et al. (2010) examined the proteome change of soybean roots under high salinity and observed that the expression of several metabolism-related proteins were mainly decreased under salt stress. Among the decreased proteins, a dienelactone hydrolase, which hydrolyzes the conversion of dienelactone to maleylacetate, which are both intermediates for the aerobic degradation of haloaromatic compounds (Schlomann et al., 1990; Blasco et al., 1995), was identified, suggesting that these secondary metabolites are not effectively degraded under salt stress. Sugimoto and Takeda (2009) performed proteomic analysis of specific proteins in root of salt-tolerant barley. In salt-tolerant barley, six proteins were identified as stress/defense-related proteins that do not scavenge ROS directly, and they had been reported in other plants, indicating that in course of time a common salt tolerance mechanism might have developed in plants.

## Root tip proteomic analysis under abiotic stress

Root is the main organ for water and nutrient absorption, and for anchorage of the plant. Longitudinally, root can be divided into three different regions: those of cell division, elongation, and maturation (Howell, 1998). The region of cell division, the root tip, contains the root apical meristem, where cells divide, but do not elongate immediately. Cell elongation and maturation in the root are thought to be controlled by the extensibility of the cell wall and the turgor pressure inside the cell (Cosgrove, 1996). The tip portion of the primary roots is important for seedling establishment (Drew et al., 1994). Proteomics analysis of the root apex revealed that the most abundant proteins are involved in stress response, glycolysis, redox homeostasis, and protein processing (Mathesius et al., 2011). All of the identified proteins of root apex were also reported to be present in differentiated root zones, but with different abundance. Flooding induced cell death in the 5-mm-long root tip region and suppressed root elongation were reported in flooded soybean seedlings (Nanjo et al., 2012).

Suppression of root elongation and induction of root tip cell death under flooding stress have also been reported in maize and pea (Subbaiah and Sachs, 2003; Gladish et al., 2006). Proteome analysis of the root tip under flooding stress was performed in soybean seedlings using mass spectrometry (MS)-based quantitative proteomics and phosphoproteomics approaches (Nanjo et al., 2012). A number of the differentially changed proteins, including sucrose-binding protein, phosphatidylinositol-4-phosphate 5-kinases, actins, and alpha-tubulins, were expressed specifically in the root tip region under flooding stress. The increased expression of sucrose-binding proteins in flooded soybean root tips implies that sucrose accumulated in roots, a finding that was reported previously in soybean roots, including the hypocotyl (Nanjo et al., 2010). A relationship between proteolysis and flooding stress was demonstrated in the root tip using a proteomics approach (Yanagawa and Komatsu, 2012). The study findings indicated that proteolytic processes involving ubiquitin/proteasomes occur in roots under flooding stress, leading to degradation of root tip cells and death of the root cap cells. Furthermore, it was demonstrated that flooding, not the hypoxic conditions, were responsible for the root tip degradation resulting from ubiquitin/proteasome-mediated proteolysis, as these injuries were independent of the oxygen concentration (Yanagawa and Komatsu, 2012).

When considering the changes in the protein abundance induced by flooding in the root tip compared to those in the root and hypocotyl, it can be inferred that the abundance of various proteins was specific to the root tip. In particular, the abundance of actins and alpha-tubulins, which are involved in cytoskeleton remodeling, was observed in only the root tip. The decrease of these proteins in the root tip suggested that they play a role in the suppression of root elongation during flooding (Nanjo et al., 2012). Further, it is clear that plant adaptation to flooding stress has involved the change of proteins in specific regions of the root, leading to the inhibition or acceleration of various biological processes, which collectively enables plants to cope with flooding stress. Proteins overrepresented in the root apex were primarily involved in the pathways for protein synthesis and processing, cell redox homeostasis, and flavonoid biosynthesis, while underrepresented proteins were those involved in glycolysis, tricarboxylic acid metabolism, and stress response. These results highlight the importance of stress and defense responses, redox control, and flavonoid metabolism in the root apex (Mathesius et al., 2011).

## Differential changes in common proteins among roots, hypocotyls and leaves under abiotic stress

**Figure 1:**
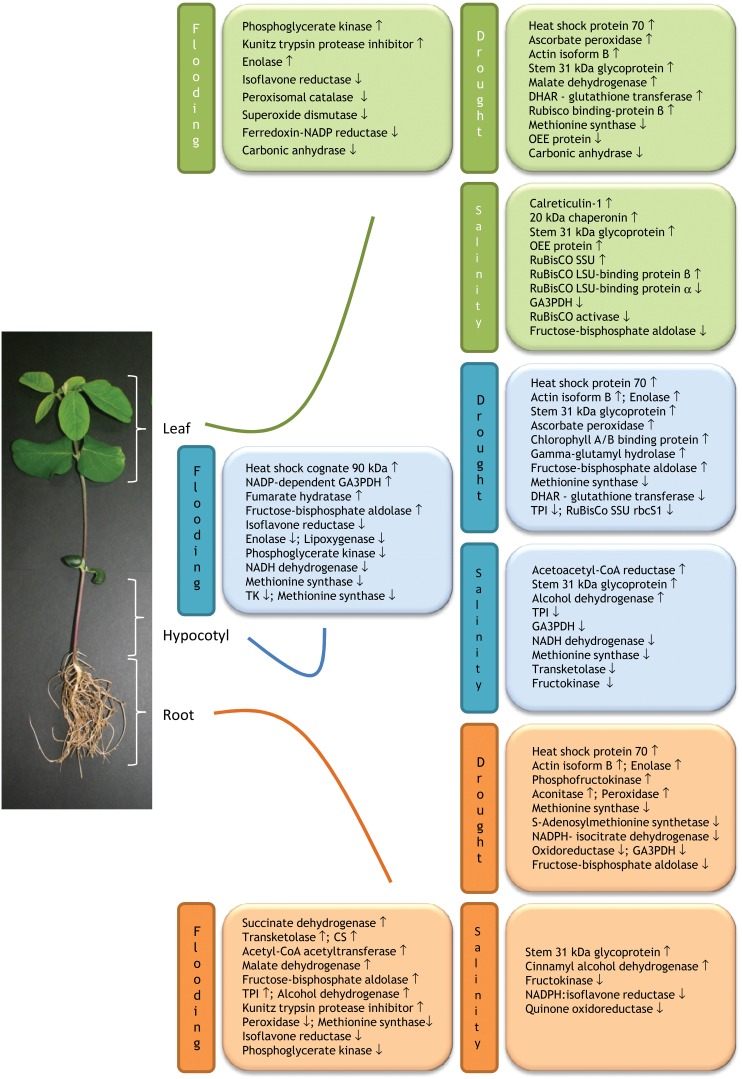
**Schematic illustration of organ-specific expression of proteins in response to drought, flooding, and salinity stresses.** The scheme is based on the published organ-specific proteomic works on soybean (*Glycine max* L.) under the mentioned abiotic stresses. Up and down arrows indicate stress-induced increase and decrease protein abundance, respectively. Abbreviations: CS, Chalcone synthase; DHAR, dehydroascorbate reductase; GA3PDH, glyceraldehyde-3-phosphate dehydrogenase; LSU, large subunit; OEE, oxygen-evolving enhancer; SSU, small subunit; TK, Transketolase; TPI, Triosephosphate isomerase.

Proteins that are commonly expressed in different plant organs under stress provide valuable information for designing genetically engineered stress tolerant crop plants. Comparison of organ-specific proteome changes on exposure to flooding stress has been well-documented in soybean. Khatoon et al. (2012) analyzed the flood induced changes in protein species using an organ-specific proteomics approach. The 2-DE separation of extracted proteins from roots, hypocotyls and leaves of 2-day-old soybeans subjected to flooding, revealed significant changes in 51, 66, and 51 protein species, respectively. In all three organs, proteins primarily involved in energy, metabolism, and disease defense showed altered expression. Among the differentially changed proteins, abundance of isoflavone reductase was commonly decreased in roots, hypocotyls and leaves under flooding stress (Figure 1). Interestingly, expression of phosphoglycerate kinase was decreased both in roots and hypocotyls while increased in leaves. Out of the four common protein species between root and hypocotyl, phosphoglycerate kinase and methionine synthase were decreased; while fructose-bisphosphate aldolase was increased under submergence. Elongation factor 1-delta protein expression was decreased and increased in root and hypocotyl, respectively. Interestingly, in both roots and leaves, Kunitz trypsin protease inhibitor was increased under flooding. In addition, Enolase was decreased in both hypocotyls and leaves in response to flooding. The remaining proteins exhibited organ-specific changes in abundance under flooding, which suggest that response mechanism of each organ in soybean seedlings is differently affected by flooding stress (Figure 1). At transcript level isoflavone reductase gene was up-regulated in leaves; while at protein level it showed decreased abundance. The difference in abundance of leaf isoflavone reductase between transcript and protein levels indicates that flooding stress affects the process of protein turnover in flooded seedlings. Their findings suggest that flooding stress lead to imbalance in expression of isoflavone reductase, involved in the biosynthesis of plant defense metabolites- lignins and isoflavonoids, along with other metabolism and disease/defense related proteins, might impair the growth of various organs in soybean seedlings under flooding stress.

Changes in protein levels in drought and PEG induced osmotic stressed soybean seedlings were analyzed using similar organ-specific proteomics approach (Mohammadi et al., 2012a,b). Among the 3 organs, root was found to be the most drought-responsive organ, with 32, 13, and 12 proteins with changed abundance in response to drought stress, PEG treatment, and both, respectively. In leaves abundances of metabolism-related proteins were increased while energy production- and protein synthesis related proteins were decreased. Findings revealed that a total of 3 proteins were commonly expressed in leaves, hypocotyls and roots of drought stressed soybean seedlings. Heat shock protein 70 and actin isoform B were upregulated and methionine synthase was downregulated under drought irrespective of the organ type (Figure 1). The observed downregulation of mRNA and declined protein levels of methionine synthase in leaves, hypocotyls, and roots of drought-stressed plants, but not in response to heat and salinity treatments indicate that methionine synthase is a drought responsive protein. All these findings suggest that the low abundance of methionine synthase might be responsible for poor growth of soybean seedlings under drought stress.

Sobhanian et al. (2010) similarly exploited proteomic techniques to unravel the effects of salt stress on organ-specific protein abundance in soybean. Exposure to 40 mM NaCl stress resulted alterations in 19, 22, and 14 protein abundances in the leaves, hypocotyls and roots, respectively. In all three organs, metabolism, and energy related proteins were largely down-regulated in response to salinity (Figure 1). Notably, glyceraldehyde-3-phosphate dehydrogenase and fructokinase were down-regulated in leaves/hypocotyls and hypocotyls/roots, respectively. In contrast, stem 31 kDa glycoprotein precursor was up-regulated in all three organs with NaCl treatment.

Taken together, all these organ-specific proteomics findings indicate that metabolism- and energy-related proteins in addition to defense play pivotal roles in each organ for adaptation to adverse environmental conditions.

## Challenges and future prospects

The present review outlines the impact of abiotic stresses on organ-specific proteome constituents. As the correlation between the expression of mRNAs and the abundance of their corresponding proteins is difficult to assess in specific organs, proteomics techniques provide one of the best options for the functional analysis of translated regions of the genome. Analysis of changes in organ-specific protein abundance strengthens our knowledge for better understanding the response mechanisms of plants to abiotic stress. Most of the investigations done so far primarily highlighted the over all organ response against a given stress. However, to pin point the stress response mechanisms of plants, changes in proteome compositions in the specific target areas within the whole organ, especially in case of roots need to be explored further.

Poor proteome coverage is often considered as the main bottleneck of organ proteomic study. While studying leaf proteome, changes in stress-responsive proteins are difficult to detect on 2-DE gel due to the presence of an abundant protein RuBisCO LSU, which accounts for about 50% of the total proteins. Fractionation of crude protein extract is the most promising technique to reach better proteome coverage. Use of anti-RuBisCO LSU antibody-affinity column to trap RuBisCO LSU protein has been an effective method to make the protein extract free of RuBisCO LSU (Hashimoto and Komatsu, 2007). This technique allowed the authors to identify four additional cold-responsive proteins in leaves of rice seedlings. More initiatives in the advancement of fractionation technique need to be taken to cope with the limited proteome resolution.

Study of changes in organ-specific proteome composition by conventional 2-DE approach coupled with MS provides a broad idea about plant stress response mechanism. However, to dissect the stress responsive biochemical pathways, targeted approaches need to be explored further. Identification of low abundance of signaling proteins and transcription factors, their protein complexes is often a challenge for classical 2-DE based organ proteomic technique. Smaczniak et al. (2012) recently reported a sensitive, quantitative proteomics based procedure to determine the composition of plant protein complexes. Fluorophore-tagged protein immunoprecipitation and label-free MS-based quantification techniques facilitate identification of low abundance signaling and regulatory protein complexes from native plant tissues. In addition, advanced technique like laser-capture micro-dissection (Dembinsky et al., 2007) for tissue proteomics could be exploited further to facilitate identification of tissue- and cell-specific proteins involved in plant responses to abiotic stress.

In systems biology, there has always been a growing demand of an accurate quantification of target sets of proteins across multiple samples (Picotti et al., 2009). Selected reaction monitoring (SRM), the most emerging targeted and highly sensitive MS technique, has great potential for the reliable identification and accurate quantitation of very low-abundance proteins in complex biological mixtures and characterization of modified peptides (Calvo et al., 2011; Picotti and Aebersold, 2012). Statistical and computational tools are essential for designing and analysis of SRM experiments, particularly in analyses of large sample (Chang et al., 2012). Picotti et al. (2009) demonstrated the potential of SRM-based proteomics technique by the consistent and fast measurement of a network of proteins spanning the entire abundance range over a growth time course of *S. cerevisiae* transiting through a series of metabolic phases. This targeted proteomics approach facilitated the detection and quantification of low abundance proteins expressed to a concentration below 50 copies/cell in total *S. cerevisiae* digests. Very recently, Picotti et al. (2013) exploited a strategy based on high-throughput peptide synthesis and MS to generate an almost complete reference map (97% of the genome-predicted proteins) of the *S. cerevisiae* proteome. The low-copy-number proteins (<103 copies/cell) play key biological roles in regulation of cellular processes or signal transduction. To elucidate a complex protein network pathway, each individual minor constituent protein needs to be identified with accuracy. The outstanding multiplexing abilities, reproducibility, sensitivity and selectivity make SRM an invaluable tool in targeted proteomics for determining very subtle expression changes, thus facilitates protein network modeling.

Improvements in multiple reaction monitoring (MRM) MS technique provide new insights into plant stress signaling pathways. Rainteau et al. (2012) used MS in the MRM mode to analyze the fatty acid composition of the major glycerophospholipids in Arabidopsis suspension cells. Findings reveal that phospholipases D action in response to salicylic acid is not due to the production of a stress-specific molecular species, but that the level of phospholipases D products *per se* is important. The over-representation of two species in phospholipases D products compared to putative substrates was linked to a regulatory role of the heterogeneous distribution of glycerophospholipids in membrane sub-domains (Rainteau et al., 2012). It is suggested that MRM-MS strategy constitutes a reliable method for determining the extract composition of glycerophospholipids in plants, essential to unravel the cell stress signaling pathway.

All these new findings indicate that, in coming days, amalgamation of diverse MS techniques coupled with bioinformatics technology with improved sample preparation and fractionation strategies would provide us more precise and comprehensive picture about plants stress response mechanism. In conclusion, more attention need to be paid in future organ-specific proteomic research to unravel those target proteins that are commonly expressed in most organs under wide range of abiotic stresses. The findings could shed some light on the cross talk between different abiotic stress signal pathways. All these valuable information would further enable us to design genetically engineered stress tolerant crop plants.

### Conflict of interest statement

The authors declare that the research was conducted in the absence of any commercial or financial relationships that could be construed as a potential conflict of interest.

## Abbreviations

RuBisCO: ribulose-1,5-bisphosphate carboxylase oxygenase
2-DE: two-dimensional polyacrylamide gel electrophoresis
TCA: trichloroacetic acid
ROS: reactive oxygen species
MS: mass spectrometry
MRM: multiple reaction monitoring.
